# Predictive factors for surgical interventions following intravenous glucocorticoid pulse therapy in active moderate-to-severe thyroid eye disease

**DOI:** 10.1007/s42000-025-00703-w

**Published:** 2025-08-06

**Authors:** Anna Giannakogeorgou, Dominik Spira, Lukas Maurer, Linus Haberbosch, Knut Mai, Daniel J. Salchow, Thomas Bobbert

**Affiliations:** 1 Department of Endocrinology and Metabolic Diseases (including Division of Lipid Metabolism), European Reference Network on Rare Endocrine Diseases (ENDO-ERN), Charité - Universitätsmedizin Berlin, corporate member of Freie Universität Berlin and Humboldt-Universität zu Berlin, Augustenburger Platz 1, 13353 Berlin, Germany; 2 Department of Ophthalmology, DRK-Kliniken Berlin, Spandauer Damm 130, 14050 Berlin, Germany; 3 Department of Ophthalmology, Charité - Universitätsmedizin Berlin, corporate Member of Freie Universität Berlin and Humboldt-Universität zu Berlin, Augustenburger Platz 1, 13353 Berlin, Germany

**Keywords:** Thyroid eye disease, Grave’s orbitopathy, Grave’s hyperthyroidism, Glucocorticoids, Intravenous methylprednisolone, Autoimmunity, Thyroid, Hyperthyroidism

## Abstract

**Purpose:**

Thyroid eye disease (TED) is the most common extrathyroidal manifestation of Graves’ disease (GD), driven by stimulatory thyrotropin receptor autoantibodies (TRAbs). Intravenous glucocorticoid pulse therapy (ivGC) is the first-line treatment for active moderate-to-severe TED. This retrospective cohort study aimed to identify predictive factors associated with treatment response in TED patients receiving ivGC therapy.

**Methods:**

We analyzed data from 146 TED patients treated with ivGC at our endocrine outpatient center between 2014 and 2021. The median treatment duration was 11 weeks (IQR: 1), with a median cumulative dose of 4.25 g (IQR: 0.25). The primary outcome was defined as the absence of the need for additional orbital decompression or strabismus surgery. Secondary outcomes included changes in ophthalmological and thyroid-related parameters. Predictive factors were identified using binary logistic regression.

**Results:**

The primary outcome was achieved in 20.54% of the patients. Predictors of surgery following ivGC treatment included current smoking (OR = 4.854, 95% CI: 1.522–15.482), age (OR = 1.051, 95% CI: 1.002–1.101), baseline diplopia (OR = 4.987, 95% CI: 1.218–20.417), and impaired visual acuity (OR = 0.274, 95% CI: 0.078–0.965). IvGC treatment improved diplopia (risk ratio = 0.87), and clinical activity score (Cohen’s d = 1.30), with a trend toward reduced TRAb levels (Cohen’s d = 0.71).

**Conclusion:**

Smoking, diplopia, and age were unfavorable for avoiding surgery, while impaired visual acuity was favorable. IvGC was associated with significant ophthalmological improvements. These findings support more personalized approaches to TED treatment.

**Supplementary Information:**

The online version contains supplementary material available at 10.1007/s42000-025-00703-w.

## Introduction

Thyroid eye disease (TED) is the most frequent extrathyroidal manifestation of Graves’ disease (GD), a prevalent autoimmune disease caused by stimulatory autoantibodies (TRAb) directed against the thyrotropin receptor (TSH-R), leading to overt hyperthyroidism and subsequent metabolic abnormalities in peripheral tissues [1]. Less frequently, TED may occur in patients with chronic autoimmune thyroiditis [2] or even patients without thyroid dysfunction [2].

TED manifests in 25–50% of people with GD [3], with the majority of cases being mild and non-progressive. Moderate-to-severe TED occurs in 5–6% of cases [4]. The global prevalence of TED in GD is approximately 40% [5]. TED typically occurs after the onset of GD with most patients having no ocular involvement at the time of diagnosis [3].

TED risk factors can be divided into nonmodifiable, modifiable, and potentially modifiable [3]. Nonmodifiable risk factors for TED include older age [3] and female sex [6], although males tend to experience more severe forms of the disease [3]. Genetic factors also play a role, with a number of identified susceptibility genes [7, 8]. Smoking, with an odds ratio (OR) of 7.7, represents the most influential modifiable risk factor, significantly increasing the likelihood and severity of TED through interfering with complex underlying autoimmune and inflammatory processes [9]. Smoking also reduces the response to immunosuppressive therapy for TED [10]. Cessation of smoking diminishes the risk of developing proptosis and diplopia, highlighting the relevance of current smoking versus lifetime exposure to tobacco [3]. The maintenance of euthyroidism is a crucial modifiable risk factor, as both hyper- and hypothyroidism adversely affect TED [4]. Elevated serum TRAb levels represent another modifiable risk factor [3]. Patients’ lipid profiles also contribute, with total and LDL cholesterol correlating with TED occurrence and severity [3]. Notably, statins among lipid-lowering drugs were associated with reductions in TED occurrence, extraocular muscle (EOM) involvement, and the need for rehabilitative surgery [3, 11]. Whether these effects are based on their anti-inflammatory or lipid-lowering effects remains unclear [3, 9].

Moderate-to-severe TED is characterized by proptosis, eyelid retraction, periorbital edema, and EOM involvement, subsequently leading to significant functional impairment due to diplopia, reduced visual acuity, and potentially dysthyroid optic neuropathy (DON) [4]. Disease activity is evaluated with the standardized clinical activity score (CAS), which examines classical signs of inflammation [4] allowing differentiation between active and inactive disease [4]. The European Group on Graves’ Orbitopathy (EUGOGO) Classification assesses TED severity as mild, moderate-to-severe, or sight-threatening based on clinical symptoms and quality of life impairment [4].

The recommended first-line treatment option for active moderate-to-severe TED by the EUGOGO) [4] involves a combination of intravenous methylprednisolone (MP) pulse therapy and the non-steroidal immunosuppressive mycophenolate sodium. However, according to the EUGOGO and American Thyroid Association (ATA) consensus statement, this treatment regimen cannot be supported due to insufficient and conflicting evidence [12]. The most frequently utilized treatment protocol remains that tested by Kahaly et al. [13] involving the administration of 0.5 g MP weekly for 6 weeks, followed by 0.25 g weekly for the following 6 weeks [4], resulting in the established optimal dose of MP for single therapy of 4.5 g.

In active TED, decompression surgery is indicated for severe exposure keratopathy, and as a second-line treatment for DON unresponsive to ivGCs [4], alleviating proptosis and reducing intraorbital pressure, with improved venous drainage [12]. During the inactive phase, residual disfigurements can be alleviated through a combination of decompression, ophthalmic plastic, and strabismus surgeries [4].

In our retrospective cohort study, our primary objective was to identify markers that predict a favorable response to ivGC pulse therapy in individuals with TED, enabling a more selective and personalized treatment approach. We aimed to support clinical decision-making by identifying patients who may require alternative or second-line treatments, minimizing unnecessary glucocorticoid exposure and side effects.

## Methods

### Study design

This retrospective cohort study was conducted by the Department of Endocrinology and Metabolism in collaboration with the Department of Ophthalmology at Charité - Universitätsmedizin Berlin, Germany. The study period spanned from January 2014 to April 2021. Clinical data were extracted from the clinical information system i.s.h.med (SAP, Walldorf, Germany) and partly via the electronic health record TBase (https://pubmed.ncbi.nlm.nih.gov/33938875/). The current study was conducted in accordance with the STROBE (Strengthening the Reporting of Observational Studies in Epidemiology) guidelines for reporting observational studies.

### Patients

The study included patients diagnosed with moderate-to-severe TED according to the EUGOGO classification with underlying GD. Exclusion criteria were other causes of exophthalmos, orbitopathy, and/or orbital deformations, a history of prior ivGC treatment, previous orbital or strabismus surgery, and therapy with oral GCs for conditions unrelated to TED. Prior oral GC treatment for TED was not an exclusion criterion.

### Treatment protocol

Patients received ivGC pulse therapy according to the standardized treatment protocol [4], recommended as first-line therapy for active, moderate-to-severe TED by the EUGOGO [4] and the ATA [12], at the Department of Endocrinology and Metabolism. Patients were started on MP at a dose of 0.5 mg MP once weekly for a total of 6 weeks, followed by 0.25 once weekly for another 6 weeks, resulting in a planned cumulative dose of 4.5 mg [4]. Ophthalmological assessments were conducted at the Department of Ophthalmology before and after completion of the treatment regimen. These assessments included measurements of visual acuity using Snellen charts or equivalent methods, evaluations of clinical signs and appearance of TED, assessment of the presence or absence of diplopia, measurement of proptosis using a Hertel exophthalmometer, and assessment of the CAS. Visual field analysis was performed using static (tendency-oriented) 30°-perimetry with the Octopus^®^ 900 instrument (Haag-Streit, Switzerland) if deemed necessary. Regular blood examinations were performed during the treatment period to avoid possible complications of steroid treatment. Additionally, all patients received lifestyle advice from the treating physician. Smoking cessation, if applicable, was strongly encouraged. From an endocrinological standpoint, maintaining euthyroidism was prioritized and treatment adjustments were made as needed.

### Statistical analysis

Descriptive statistics were calculated for baseline characteristics. To assess data normality, the Shapiro-Wilk and Kolmogorov-Smirnov tests were used. For therapy efficacy, appropriate statistical tests were applied to compare means before and after treatment, depending on data distribution. A Bonferroni correction was applied to control the overall error rate. Stepwise logistic regression identified significant predictive factors for therapy efficacy. To assess the overall significance of the model, we utilized the omnibus test, along with individual p-values to evaluate the significance of each predictor and OR to interpret the magnitude and direction of the relationships between predictors and the outcome. To address class imbalance in the original logistic regression model, we applied the synthetic minority over-sampling technique (SMOTE) to oversample the minority class. All statistical analyses were performed using IBM SPSS Statistics version 29.0 (142), while RStudio version 2023.12.1 + 402 (2023.12.1 + 402) was utilized for SMOTE using the “smotefamily” package. Significance levels were set at *p* < 0.05.

### Outcomes

#### Primary outcome

The primary outcome of this study was the identification of prognostic factors associated with the need for further surgical interventions, such as orbital decompression or strabismus surgery, following ivGC pulse therapy in patients with active, moderate-to-severe TED.

#### Secondary outcomes

Secondary outcomes were improvement of ophthalmological parameters and symptoms as well as thyroid hormone and antibody levels.

### Ethical considerations

Approval for the retrospective study “Kahaly Scheme in Endocrine Orbitopathy” was obtained from the Ethics Committee at Charité– University Hospital Berlin (EA4/170/21). The study protocol, including participant recruitment, data collection, and informed consent, was approved and complies with the 1964 Declaration of Helsinki and its amendments.

## Results

### Patients

A total of 146 patients, who completed the 12-week regimen, were included in this study. Baseline demographic characteristics of the study population are presented in Table 1.

**Table 1:** Baseline demographic characteristics of the study population

Demographic characteristics	
Gender	
Male, N (%)	43 (29.5%)
Female, N (%)	103 (70.5%)
Age, y, mean ± SD	51.79 ± 13.27
BMI (kg/m^2^), median (IQR)	25.80 (22.95–30.30)
Mean blood pressure	
Systolic, mmHg, median (IQR)	130.0 (120–145)
Diastolic, mmHg, median (IQR)	79.5 (74–86)
Smoking status	
Smoker, N (%)	49 (33.6%)
Non-smoker, N (%)	97 (66.4%)
Pack years among ever-smokers, y, median (IQR)	30 (15–40)
*Baseline thyroid biochemistry (reference range)*	
TSH (0.27–4.2 mU/L) median (IQR)	1.11 (0.16–2.41)
fT3 (2.0–4.4 ng/L) median (IQR)	3.09 (2.65–3.83)
fT4 (9.3–17.0 ng/L) mean ± SD	14.74 ± 9.4
TRAb (< 2.0 U/I) median (IQR)	7.81 (1.35–18.70)
Tg-Ab (< 115 kU/I) median (IQR)	17.5 (10.10-64.75)
TPO-Ab (≤ 35 KU/I) mean ± SD	95.1 ± 136.34
*Medication*	
ATD, N (%)	46 (31.5%)
L-thyroxine, N (%)	72 (49.3%)
Statins, N (%)	11 (7.5%)
*Supplementation*	
Selenium, N (%)	32 (21.9%)

For normally distributed data, mean and SD were used. For non-normally distributed data, median and range are shown. For thyroid hormones and antibody levels, reference ranges are given.

*Abbreviations*: ATD, antithyroid drugs; BMI, body-mass index; fT3, free triiodothyronine; fT4, free thyroxine; SD, standard deviation; TG-Ab, thyroglobulin antibodies; TPO AB, thyroid peroxidase antibodies; TRAb, thyrotropin receptor antibodies; TSH, thyroid-stimulating hormone

### Primary outcome

We investigated the ability to predict whether a patient would not require additional eye muscle or orbital decompression surgery after ivGC treatment completion. In total, 20.5% of patients required additional surgery after completing the ivGC regimen. A total of 17 patients underwent orbital decompression surgery, 10 underwent strabismus surgery, and three required both. We examined whether age, sex, current smoking status, lifetime tobacco exposure measured in pack years, diplopia, impairment of visual acuity, and presence of proptosis before treatment initiation, along with euthyroidism, could potentially act as predictors of treatment success. Euthyroidism was defined as TSH and FT4 being within the reference range of our corresponding lab, Labor Berlin (0.4–4.0 mU/L for TSH and 8–18 ng/L for fT4, respectively), while the cut-off value for TRAb positivity was 2.0 U/L. Baseline proptosis was defined as Hertel-exophthalmometry values above the cut-off based on race and sex, specifically 19 mm for female and 21 mm for male Caucasians [14]. Impairment of visual acuity was defined as any value below < 1.0 (10/10) in best corrected visual acuity (BCVA) in at least one eye.

Our analysis revealed that current smoking, older age, baseline diplopia, and impairment of visual acuity were significant predictors, with smokers having almost five times the risk (OR = 4.854, 95% confidence interval [CI] 1.522–15.482, *p* = 0.008) of requiring surgery compared to non-smokers. Additionally, patients with baseline diplopia had nearly five times the risk of needing additional surgery compared to those without diplopia (OR = 4.987, 95% CI: 1.218–20.417, *p* = 0.025). Surprisingly, patients presenting with impaired visual acuity had a reduced risk of requiring subsequent surgery (OR = 0.274, 95% CI: 0.078–0.965, *p* = 0.044). Age also emerged as a significant predictor of the need for surgery, with each additional year of age increasing the odds by 5% (OR = 1.051, 95% CI: 1.002–1.101, *p* = 0.039) (Table 2).

**Table 2:** Binary logistic regression model predicting surgical intervention probability following intravenous glucocorticoid treatment

Variable	Coefficient B	SE	*p*-value	Exp(B)	95% CI for Exp(B)	
					Lower	Upper
Smoking	1.580	0.592	0.008	4.854	1.522	15.482
Diplopia	1.607	0.719	0.025	4.987	1.218	20.417
Impairment of visual acuity	-1.294	0.642	0.044	0.274	0.078	0.965
Age	0.049	0.024	0.039	1.051	1.002	1.101
Constant	-5.069	1.493	< 0.001	0.006		

Binary logistic regression was calculated after inclusion of potential covariates. Non-significant covariates, such as biological sex, euthyroidism, TRAb positivity, baseline proptosis, and statin treatment were excluded, leaving smoking, diplopia, impairment of visual acuity, and age as contributors for the most efficient model.

*Abbreviations*: 95% CI, 95% confidence interval for odds ratio; Exp(B); odds ratio; SE, standard error

In our analysis, the binary logistic regression model demonstrated robust predictive accuracy for patients with available data for all variables included in the model who responded well to ivGC therapy and did not require additional surgery after completing the 12-week regimen, correctly classifying 94.9% of these cases (*n* = 74). For the subset of patients requiring surgery (*n* = 14), the model’s classification rate was low (26.3%), likely due to class imbalance. The model overfitted to the majority class (non-surgery), leading to poor predictions for the minority class (surgery). To address this, we applied SMOTE to create 86 synthetic samples, balancing both groups with all available variables (*n* = 116). Missing values were imputed with column means. We reran the logistic regression to improve predictive performance and reduce overfitting.

The balanced (undersampled) regression model identified two shared significant predictors with the initial model for the entire cohort, namely, smoking (OR = 3.044, 95% CI: 1.600-5.793, *p* < 0.001) and baseline diplopia (OR = 3.155, 95% CI: 1.586–6.277, *p* = 0.001). Additionally, in the balanced model, euthyroidism (OR = 2.101, 95% CI: 1.034–4.271, *p* = 0.040) and TRAb positivity (OR = 2.223, 95% CI: 0.901–5.486, *p* = 0.083) were identified as positive predictors of the need for surgery, whereas, interestingly, baseline proptosis (OR = 0.440, 95% CI: 0.226–0.858, *p* = 0.016) emerged as a negative predictor (Supplementary Table 1).

The balanced binary logistic regression model improved predictive performance, correctly identifying 66.4% of patients requiring surgery and 60.3% of treatment responders. This balanced approach enhanced the accuracy of predicting surgical intervention.

It is of interest to note that TRAb positivity was retained in the final step of the balanced model but did not reach statistical significance (*p* = 0.083), raising questions about its potential influence. Despite this, it demonstrated a large effect size. Given its relevance in the context of TED, we aimed to further explore TRAb positivity as a predictor by re-running the regression model in the balanced cohort after excluding TRAb positivity. The analysis showed that smoking, baseline diplopia, and baseline proptosis remained significant predictors even after TRAb positivity was removed from the model (Supplementary Table 2).

Across all three regression analyses, active smoking and baseline diplopia consistently predicted the need for subsequent surgery after ivGC therapy. In both the original and SMOTE-balanced cohorts, as well as after removing TRAb positivity, current smoking and baseline diplopia were linked to an approximately three-fold increase in the risk of surgery.

### Secondary outcomes

#### Ophthalmic outcomes

We compared the difference in several ophthalmic parameters before and after treatment to evaluate the efficacy of ivGC treatment in TED. We observed a statistically significant difference in diplopia before and after treatment (risk ratio = 0.87, *p* < 0.001). CAS assessments pre- and post-intervention showed statistically significant differences after ivGC therapy (Cohen’s d = 1.30, *p* < 0.001). The changes in the rest of the ophthalmic parameters did not show statistical significance (Table 3).

**Table 3:** Ophthalmological assessments at baseline and at the end of the treatment period

Variable	Baseline	Post treatment	Adjusted *p*-value (Bonferroni)	Paired measurements available (*n* (%))
***Disease activity***				
CAS, mean ± SD	3.94 ± 1.52	1.95 ± 1.428	< 0.016	34 (23.3)
***Disease severity***				
Diplopia, N (%)	95.0 (65.1%)	54.8 (56.7%)	< 0.016	146 (100)
Visual acuity RE, median (IQR)	0.8 (0.60-1.00)	1.0 (0.65-1.00)	0.064	132 (90.4)
Visual acuity LE, median (IQR)	0.8 (0.80-1.00)	1.0 (0.80-1.00)	0.112	132 (90.4)
Proptosis RE, mm, median (IQR)	20.0 (18.0-22.5)	20.0 (18.0–22.0)	0.528	138 (94.5)
Proptosis LE, mm, median (IQR)	21.0 (18.5–23.0)	20.0 (19.0–22.0)	0.768	122 (83.7)
***Visual field examination***				
MD RE, dB, median (IQR)	-3.40 (-6.05,-1.35)	-2.40 (-3.80,-0.70)	0.544	77 (52.7)
MD LE, dB, median (IQR)	-3.25 (-5.95,-1.25)	-2.45 (-4.90,-1.00)	1.000	77 (52.7)
SRLV RE, dB, median (IQR)	2.8 (2.0-3.8)	2.4 (1.9–3.4)	1.000	77 (52.7)
SRLV LE, dB, median (IQR)	2.3 (1.9–3.8)	2.5 (2.0-3.6)	1.000	77 (52.7)

Summary of ophthalmological parameters before and after intravenous glucocorticoid (ivGC) treatment. Mean and standard deviation (SD) are presented for normally distributed data, while median and interquartile range (IQR) are shown for non-normally distributed data. The last column indicates the number of patients with paired measurements available for each variable before and after treatment out of the total cohort of 146 patients. Significance levels were adjusted for multiple testing using the Bonferroni correction, with a corrected p-value threshold of 0.003 P-values adjusted for multiple testing greater than 1 are capped at 1.000.

*Abbreviations*: CAS, clinical activity score; ivGC, intravenous glucocorticoids; LE, left eye; MD, mean difference; RE, right eye; SRLV, square root of loss variance

Despite the overall improvements in clinical TED parameters, our findings revealed a counterintuitive exacerbation of orbital manifestations in some individuals. In our cohort, 30.1% of patients experienced worsened proptosis and 21.9% demonstrated worsening of visual acuity in at least one eye following treatment. We identified baseline diplopia and presence of obesity as predictors of worsened proptosis in either eye. Specifically, obesity was associated with a more than 10-fold increased risk of proptosis worsening (OR = 12.161, 95% CI: 1.334-110.828). Baseline diplopia was associated with a reduced risk of proptosis deterioration (OR = 0.047, 95% CI: 0.005–0.404) (Supplementary Table 3). Age was identified as a predictor of de novo development of diplopia during ivGC treatment (OR = 1.065, 95% CI: 1.005–1.130; Supplementary Table 4), with each 1-year increase in age associated with a 6.5% increase in risk (Supplementary Table 4).

We performed additional analyses comparing ophthalmological parameters before and after ivGC treatment in the surgical (*n* = 30) and non-surgical (*n* = 116) groups. Interestingly, significant improvements in CAS and diplopia were observed only in the non-surgical group (*p* < 0.001 for both) (Supplementary Table 5).

### Thyroid hormones and antibodies

Regarding thyroid markers, the differences in all measured parameters did not reach statistical significance. TRAb levels showed a decreasing trend, albeit not statistically significant (Cohen’s d = 0.71, *p* = 0.012) (Table 4). We observed no differences in thyroid hormone or antibody levels between patients who underwent surgery and those who did not, either before or after ivGC therapy (Supplementary Table 6).

**Table 4:** Thyroid hormone and antibody levels before and after treatment

Variable	Baseline	Post treatment	Adjusted *p*-value (Bonferroni)	Paired measurements available (*n* (%))
TSH (mU/L), median (IQR)	1.44 (0.17–2.32)	2.04 (0.82–2.90)	1.000	79 (54.1)
fT3 (pg/mL), median (IQR)	3.01 (2.68–3.51)	3.17 (2.84–3.56)	1.000	44 (30.13)
fT4 (ng/L), median (IQR)	13.50 (10.79–15.40)	13.80 (12.00-16.20)	1.552	62 (42.5)
TRAbs (U/l), median (IQR)	6.97 (0.89–13.19)	2.29 (0.89–5.75)	0.192	12 (8.2)
TPO-Ab (kU/l), mean ± SD	20.83 ± 17.71	34.00 ± 38.22	1.000	6 (4.1)
Tg-Ab (kU/l), median (IQR)	14.05 (11.60-32.35)	14.85 (11.95–187.90)	1.000	5 (3.4)

Summary of thyroid hormones and antibodies before and after intravenous glucocorticoid (ivGC) treatment. Mean and standard deviation (SD) are presented for normally distributed data, while median and interquartile range (IQR) are shown for non-normally distributed data. The last column indicates the number of patients with paired measurements available for each variable before and after treatment out of the total cohort of 146 patients. Significance levels were adjusted for multiple testing using the Bonferroni correction, with a corrected p-value threshold of 0.003. Bonferroni-adjusted p-values greater than 1 are capped at 1.000.

### Safety / adverse events

None of the 146 patients developed DON or experienced glucocorticoid-associated adverse effects during or after treatment.

## Discussion

It is noteworthy that one of the strongest predictive factors for treatment success that we identified across all regression models, namely, current smoking, is largely modifiable. Based on our results, younger, non-smoking patients without diplopia presenting with impairment of visual acuity are less likely to require orbital decompression or strabismus surgery following ivGC therapy. Conversely, TED patients who do not meet these criteria are more likely to require surgery. These insights may help optimize TED treatment and guide personalized therapy based on baseline phenotype.

We found an almost fivefold increase in the likelihood of surgery after ivGC treatment for individuals engaging in smoking, in alignment with previous research showing that nonsmokers respond better to corticosteroids [3]. It is pertinent to note that our study highlights current smoking status as a strong predictor of therapy efficacy and the necessity for orbital decompression or strabismus surgery, while pack years and past smoking history are considered insignificant. Importantly, diplopia and proptosis, key TED symptoms affecting visual function and quality of life, appear highly influenced by smoking status [15]. Diplopia is a known risk factor for sight-threatening disease or progression to DON, underscoring the need for comprehensive strategies targeting specific symptoms to enhance patient outcomes [12]. Screening for diplopia at the time of GD diagnosis could therefore be beneficial. Additionally, older age was associated with an increased risk for surgery, confirming previous findings showing poorer responses to glucocorticoids and the need for further treatment measures among older individuals [2, 16]. Surprisingly, we found that patients with impairment of visual acuity at baseline were less likely to require surgery compared to patients with unaffected visual acuity. This could be explained by earlier treatment initiation prompted by the urgent nature of the symptom. Of note, visual acuity impairment in TED is predominantly associated with inflammatory processes, such as optic nerve compression, which may be more responsive to ivGC therapy [4]. In contrast, symptoms such as diplopia and proptosis can result from both inflammatory and fibrotic changes, making them less responsive to steroid treatment [17] and necessitating surgical interventions, regardless of responsiveness to glucocorticoids.

In the SMOTE-balanced cohort, diplopia and smoking remained significant predictors of surgery. In addition, baseline proptosis emerged as a negative predictor of the need for additional surgery, in contrast with previous findings suggesting that ivGC therapy has limited effectiveness in addressing proptosis and that patients with significant baseline proptosis might be more likely to require surgery [18]. This discrepancy may be explained by the characteristics of our study population. Notably, almost one third (34.4%) of our cohort did not have proptosis at baseline [14]. Among patients with milder proptosis, modest improvements from ivGC therapy could suffice to avoid additional surgery. Further research is needed to clarify the relationship between the degree of baseline proptosis and the need for surgical intervention across diverse populations and in patients with more significant proptosis. Moreover, the unexpected finding of euthyroidism as a predictor of subsequent surgery may be due to limitations in our thyroid function assessment, since the model included only baseline thyroid function without taking into consideration potential fluctuations during treatment. TRAb positivity initially showed marginal significance as a predictor of the need for surgery but was ultimately removed from the model. After its exclusion, only baseline diplopia and smoking remained as consistent predictive factors across all regression models. While TRAb positivity could have influenced thyroid status, our assays did not differentiate between blocking and stimulating TRAbs [19].

We found no association between concomitant treatment with statins during ivGC treatment and the need for orbital decompression or strabismus surgery. In the Statins for Grave’s Orbitopathy (STAGO) trial, atorvastatin as an adjunct to ivGCs in hypercholesterolemic TED patients led to improvements in ophthalmic symptoms and reductions in relapse rate [20]. Of note, no differences in LDL cholesterol concentration between responders and non-responders to methylprednisolone were observed suggesting that the effects of statins on TED are unrelated to their lipid-lowering actions [11, 20] and could be related to their autophagy- and apoptosis-modulating abilities [21]. Recent research showed that simvastatin mitigates upregulated inflammatory gene expression as well as B and T cell proliferation in peripheral blood mononuclear cells (PBMCs) from GD patients after exposure to cigarette smoke extract, revealing new mechanisms of the effects of statins in the context of TED [9]. It is important to note that most available studies examining various immunomodulatory agents in TED used the CAS scoring system to determine therapeutic outcome. Our study used the need for orbital decompression or strabismus surgery as an outcome. This outcome is in line with patients’ perspective on treatment success, focusing on the resolution of symptoms and improvement in quality of life [22]. Since baseline CAS scores had not been evaluated for the majority of the study participants, using this binary endpoint bypasses the limitations associated with CAS assessments, such as interobserver variability and potential subjectivity [12]. In our cohort, using overall reduction in proptosis or improvement in visual acuity as the primary endpoint would be impractical, as 42.8% of participants presented with asymmetric disease and significant proptosis and visual acuity were not present at baseline. However, it should be noted that the present study may have been underpowered to detect such an effect. Larger studies are required to investigate the effect of statin treatment during ivGC on the need for additional surgery in TED.

Despite overall improvements in clinical TED parameters, some patients experienced exacerbation of orbital manifestations, specifically, worsening of visual acuity and proptosis. Obesity was identified as a positive predictor of worsening of proptosis following ivGC therapy, in alignment with previous research linking obesity with TED. Proposed mechanisms are chronic low-grade inflammation present in obesity [23], which may exacerbate the autoimmune processes in TED [24, 25] and increased adipogenesis [26] contributing to orbital fat expansion and proptosis, as well as altered pharmacokinetics of steroids, potentially reducing their effectiveness in TED. While obesity was associated with deterioration of proptosis, the wide 95% CI indicates substantial uncertainty in this estimate and suggests the need for further investigation with a larger sample size. Interestingly, baseline diplopia was identified as a negative predictor for deterioration of proptosis. This could be explained by differential tissue response to steroids, as the presence of diplopia indicates predominant extraocular muscle involvement rather than orbital adipose tissue expansion, which is more predominant in proptosis [27]. Additionally, presence of diplopia serves as a major indication for therapy and is indicative of more advanced TED [28]. Laurberg et al. also found that diplopia correlates negatively with proptosis, this according with our findings [28].

We found no significant improvements in visual acuity before and after treatment, possibly due to less pronounced baseline reductions compared to other TED symptoms like diplopia and proptosis. It is noteworthy that 32.2% of patients had no TED-related visual acuity impairment at baseline. Previous studies have yielded inconclusive results regarding the effect of ivGC treatment on TRAb levels. While some studies observed significant reductions in TRAb levels following ivGC treatment [29, 30], we found no such reduction, similarly to the findings of other studies [20]. Lower baseline concentrations of TRAbs have previously been linked to better response to immunosuppressive treatment [29, 31]. In our cohort, TRAb levels were not identified as a predictor of surgical intervention following ivGC treatment.

Our study has several strengths. First, in addition to standard clinical TED-related parameters we incorporated visual field examinations, providing a more comprehensive evaluation of orbital pathology. Moreover, the relative uniformity in examinations conducted at one specialized center ensured consistency and reliability in outcome assessments.

While our study offers valuable insights, several limitations should be noted. The small sample size may affect the generalizability of our findings. Furthermore, the retrospective design introduces inherent challenges, while the lack of a control group limits our ability to establish causal relationships. The use of a binary variable for diplopia instead of the Gorman Score may reduce assessment precision. Additionally, the study period overlapped with the COVID-19 pandemic, potentially leading to incomplete follow-up data, particularly regarding perimetry examinations, CAS and thyroid antibodies and hormones. Finally, the use of SMOTE to balance the dataset may cause underfitting and limit generalizability. Future studies should incorporate more factors and a larger cohort to enhance predictive accuracy for surgical intervention following ivGC therapy.

## Conclusion

Patients with moderate-to-severe TED are heterogeneous at clinical presentation and exhibit diverse responses to ivGC therapy, with divergent outcomes regarding the necessity for surgical intervention. We identified smoking, diplopia, and older age as predictors associated with an increased likelihood of requiring surgical intervention, whereas impaired visual acuity at baseline was associated with a reduced likelihood.

Based on baseline ophthalmic features, thyroid function, and antibodies, patients may be stratified into clinically meaningful subgroups thus tailoring a more individualized therapeutic approach.

The presented findings highlight the need for personalized management strategies in TED and underscore the importance of early risk stratification. Larger, prospective studies are warranted to identify predictors of response to second-line therapies and to validate these findings across more diverse populations.

## Supplementary Information

Below is the link to the electronic supplementary material.

Supplementary Material 1

## Data Availability

The data collected for this study, including individual participant data, will not be made available to others. Given that the study participants are patients of Charité University Hospital Berlin, sharing individual-level data is restricted to protect patient confidentiality. Aggregated data or summaries may be shared upon reasonable request, in compliance with applicable data protection regulations.
