# Supplementary material for: Predictive factors for surgical interventions following intravenous glucocorticoid pulse therapy in active moderate-to-severe thyroid eye disease
Source: Hormones (Athens). 2025 Aug 6;24(4):1013–21. doi: 10.1007/s42000-025-00703-w (PMC12678594; doi:10.1007/s42000-025-00703-w)
Supplement: Supplementary file 1 — Supplementary Material 1 [file 42000_2025_703_MOESM1_ESM.docx]

**Supplementary Material**

**Table of contents**

Supplementary Table 1 ……………………………………………………………….……. 2

Supplementary Table 2 ……………………………………………………………….……. 3

Supplementary Table 3 ……………………………………………………………….……. 4

Supplementary Table 4 ……………………………………………………………….……. 5

Supplementary Table 5 ……………………………………………………………….……. 6

Supplementary Table 6 ……………………………………………………………….……. 8

**Supplementary Table 1. SMOTE-Balanced binary logistic regression model predicting surgical intervention probability following intravenous glucocorticoid treatment**

| **Variable** | **Coefficient B** | **SE** | **p-value** | **Exp(B)** | **95% CI for Exp(B)** | |
| --- | --- | --- | --- | --- | --- | --- |
|  |  |  |  |  | **Lower** | **Upper** |
| Smoking | 1·113 | 0·328 | <0·001 | 3·044 | 1·600 | 5·793 |
| Baseline Diplopia | 1·149 | 0·351 | 0·001 | 3·155 | 1·586 | 6·277 |
| Euthyroidism | 0·743 | 0·362 | 0·040 | 2·101 | 1·034 | 4·271 |
| TRAb Positivity | 0·799 | 0·461 | 0·083 | 2·223 | 0·901 | 5·486 |
| Baseline Proptosis | -0·821 | 0·341 | 0·016 | 0·440 | 0·226 | 0·858 |
| Constant | -1·741 | 0·554 | 0·002 | 0·175 |  |  |
| Binary Logistic Regression was calculated after oversampling the minority group (no surgery) via a SMOTE approach and inclusion of potential covariates in the balanced dataset. Non-significant covariates, such as biological sex, age, impairment of visual acuity, statin treatment were excluded, leaving smoking, diplopia, baseline proptosis, euthyroidism, and TRAb positivity as contributors for the most efficient model.  Abbreviations 95% CI, 95% Confidence Interval for Odds Ratio; Exp(B), Odds Ratio; SE, Standard Error. TRAb, TSH Receptor Antibodies | | | | | | |

**Supplementary Table 2. SMOTE-Balanced binary logistic regression model predicting surgical intervention probability upon removal of TRAb positivity.**

| **Variable** | **Coefficient B** | **SE** | **p-value** | **Exp(B)** | **95% CI for Exp(B)** | |
| --- | --- | --- | --- | --- | --- | --- |
|  |  |  |  |  | **Lower** | **Upper** |
| Smoking | 1·234 | 0·325 | <0·001 | 3·434 | 1·817 | 6·490 |
| Baseline Diplopia | 1·272 | 0·347 | <0·001 | 3·569 | 1·809 | 7·043 |
| Baseline Proptosis | -0·825 | 0·336 | 0·014 | 0·438 | 0·227 | 0·847 |
| Constant | -0·900 | 0·386 | 0·020 | 0·406 |  |  |
| Binary Logistic Regression was calculated after oversampling the minority group (surgery) via a SMOTE approach and inclusion of potential covariates in the balanced dataset, with the exception of TSH receptor antibodies. Non-significant covariates were excluded, leaving smoking, diplopia and baseline proptosis, as contributors for the most efficient model. | | | | | | |

**Supplementary Table 3. Binary logistic regression model predicting deterioration of proptosis following intravenous glucocorticoid pulse therapy**

| **Variable** | **Coefficient B** | **SE** | **p-value** | **Exp(B)** | **95% CI for Exp(B)** | |
| --- | --- | --- | --- | --- | --- | --- |
|  |  |  |  |  | **Lower** | **Upper** |
| Diplopia | -3ª061 | 1·099 | 0·005 | 0·047 | 0·005 | 0·404 |
| Obesity | 2·498 | 1·127 | 0·027 | 12·161 | 1·334 | 110·828 |
| Constant | -0·127 | 0·610 | 0·835 | 0·881 |  | |
| Binary Logistic Regression was calculated after inclusion of potential covariates, including smoking status TRAb positivity, euthyroidism, leaving obesity, defined as a BMI ≥ 30 kg/m^2^, and baseline diplopia as significant predictors for worsening of proptosis in at least one eye following ivGC treatment. Proptosis deterioration was defined as any measurable increase in Hertel exophthalmometry values in at least one eye. | | | | | | |

**Supplementary Table 4. Binary logistic regression model predicting deterioration of diplopia following intravenous glucocorticoid pulse therapy**

| Variable | Coefficient B | SE | p-value | Exp(B) | 95% CI for Exp(B) | |
| --- | --- | --- | --- | --- | --- | --- |
|  |  |  |  |  | Lower | Upper |
| Age | 0·063 | 0·030 | 0·035 | 1·065 | 1·005 | 1·130 |
| Constant | -4·526 | 1·688 | 0·007 | 0·011 |  | |
| Binary Logistic Regression was calculated after inclusion of potential covariates, including age, sex, BMI, smoking status, smoking history in pack years, TRAb positivity, euthyroidism and asymmetric disease presentation. Age was identified as significant predictors of deterioration of visual acuity in either eye. | | | | | | |

**Supplementary Table 5: Ophthalmological parameters in surgical and non-surgical groups before and after ivGC treatment**

| **Variable** | **Non-surgical group (n=116)** | | **p-value (within non-surgical group)** | **Surgical group (n=30)** | | **p-value (within surgical group)** | **p-value (between-groups, baseline)** | **p-value (between-groups, after ivGC)** |
| --- | --- | --- | --- | --- | --- | --- | --- | --- |
|  | **Baseline** | **After ivGC** |  | **Baseline** | **After ivGC** |  |  |  |
| ***Disease activity*** | | | | | | | | |
| **CAS, mean ± SD** | **4 ± 1** | **2 ± 1** | **<0.001** | **4 ± 2** | **3 ± 2** | **0.160** | **0.761** | **0.109** |
| ***Disease severity*** | | | | | | | | |
| **Diplopia, N (%)** | **70 (60.3)** | **55 (49.5)** | **<0.001** | **25 (93.3)** | **25 (93.3)** | **1.000** | **0.019** | **<0.001** |
| **Visual acuity RE, median (IQR)** | **0.8 (0.6-1.0)** | **1.0 (0.8-1.0)** | **0.001** | **0.8 (0.6-1.0)** | **0.8 (0.6-1.0)** | **0.928** | **0.388** | **0.142** |
| **Visual acuity LE, median (IQR)** | **0.8 (0.8-1.0)** | **1.0 (0.8-1.0)** | **0.006** | **0.8 (0.8-1.0)** | **0.8 (0.7-1.0)** | **0.634** | **0.951** | **0.312** |
| **Proptosis RE, mm, median (IQR)** | **20.0 (18.3-22.0)** | **20.0 (19.0-22.0)** | **0.088** | **20.0 (18.0-23.0)** | **19.0 (18.0-23.0)** | **0.251** | **0.965** | **0.940** |
| **Proptosis LE, mm, median (IQR)** | **21.0 (18.5-23.0)** | **20.0 (19.0-22.0)** | **0.076** | **21.0 (18.0-23.0)** | **20.0 (17.0-23.0)** | **0.370** | **0.389** | **0.595** |
| ***Visual field examination*** | | | | | | | | |
| **MD RE, dB, median (IQR)** | **-2.70 (-6.05-(-1.20))** | **-2.40 (-3.90-(-0.70))** | **0.183** | **-4.40(-5.30-(-2.90))** | **-2.16(-3.20(-1.20))** | **0.059** | **0.114** | **0.673** |
| **MD LE, dB, median (IQR)** | **-2.90 (-5.95-(-1.15))** | **-2.50 (-4.90(-1.00))** | **0.793** | **-3.30(-4.90(-3.00))** | **-2.00(-4.70-(-1.30))** | **0.069** | **0.155** | **0.852** |
| **SRLV RE, dB, median (IQR)** | **2.80 (2.10-3.60)** | **2.30 (1.90-3.30)** | **0.451** | **3.20 (2.00-4.20)** | **2.60 (2.40-4.50)** | **0.575** | **0.413** | **0.231** |
| **SRLV LE, dB, median (IQR)** | **2.30 (1.90-3.30)** | **2.30 (1.90-3.50)** | **0.788** | **2.90 (2.10-4.50)** | **3.40 (2.20-4.60)** | **0.370** | **0.152** | **0.115** |
| **Overview of ophthalmological parameters, including perimetry examination, is shown for patients who did not require surgery and those who underwent surgery following completion of ivGC treatment. Data are presented as mean ± SD for normally distributed variables and as median (IQR) for skewed parameters. Within-group comparisons before and after treatment were performed using paired t-tests or Wilcoxon signed-rank tests, depending on the distribution of the data. Between-group comparisons were conducted using the independent samples t-test or the Mann–Whitney U test, as appropriate based on skewness and distribution. For diplopia, within-group comparisons were assessed using McNemar’s test, and between-group comparisons with the Chi-square test. p-values are reported without correction for multiple comparisons, as these results are presented as supplementary data. A Bonferroni-corrected significance threshold was set at p = 0.003 for interpretative reference.** | | | | | | | | |

**Supplementary table 6. Thyroid markers in surgical vs. non-surgical patients before and after ivGC treatment**

| **Variable** | **Non-surgical group (n=116)** | | **p-value (within surgical group)** | **Surgical group (n=30)** | | **p-value (within non-surgical group)** | **p-value (between-groups, baseline)** | **p-value (between-groups, after ivGC)** |
| --- | --- | --- | --- | --- | --- | --- | --- | --- |
|  | **Baseline** | **After ivGC** |  | **Baseline** | **After ivGC** |  |  |  |
| **TSH (mU/L), median (IQR)** | **1.1 (0.2-2.5)** | **1.3 (0.6-3.0)** | **0.315** | **1.3 (0.4-2.3)** | **1.8 (0.8-2.3)** | **0.875** | **0.489** | **0.844** |
| **fT3 (pg/mL), median (IQR)** | **3.1 (2.7-3.9)** | **3.1 (2.8-3.6)** | **0.528** | **3.1 (2.6-3.7)** | **3.6 (2.8-4.2)** | **0.356** | **0.881** | **0.297** |
| **fT4 (ng/L), median (IQR)** | **13.8 (11.0-16.4)** | **14.4 (11.7-16.8)** | **0.100** | **14.5 (11.1-16.6)** | **13.4 (12.1-14.5)** | **0.815** | **0.761** | **0.522** |
| **TRAbs (U/l), median (IQR)** | **7.4 (1.0-17.2)** | **2.2 (0.9-8.5)** | **0.028** | **10.1 (2.6-23.6)** | **1.0 (0.9-1.1)*** | **0.180** | **0.130** | **0.553** |
| **TPO-Ab (kU/l), median (IQR)** | **22 (11-114)** | **14 (9-55)** | **0.465** | **84 (31-118)** | **9**** | **NA** | **0.035** | **0.604** |
| **Tg-Ab (kU/l), median (IQR)** | **15.3 (10.2-44.7)** | **14.9 (12.5-52.6)** | **0.224** | **22.6 (14.6-337.8)** | **1012**** | **NA** | **0.146** | **0.134** |
| **Comparison of thyroid hormone levels and thyroid-related antibodies before and after ivGC treatment in patients who did not require surgery and those who underwent surgery. Availability of follow-up thyroid antibody levels after ivGC treatment was limited, especially in the surgical group. Data are presented as median (IQR). Within-group comparisons before and after treatment were performed using paired t-tests or Wilcoxon signed-rank tests, depending on the distribution of the data. Between-group comparisons were conducted using the independent samples t-test or the Mann–Whitney U test, as appropriate based on skewness and distribution. p-values are reported without correction for multiple comparisons, as these results are presented as supplementary data. For interpretative purposes, the Bonferroni-adjusted significance level was set at p = 0.003.**  ***Variables available for only n=2 in the surgical group and should be interpreted with caution. No within-group comparison before and after treatment possible.**  ****Variables available for only n=1 in the surgical group and should be interpreted with caution. No average provided. No within-group comparison before and after treatment possible.** | | | | | | | | |
